# Longitudinal analysis of a secondary *BRCA2* mutation using digital droplet PCR

**DOI:** 10.1002/cjp2.146

**Published:** 2019-11-20

**Authors:** Saira Khalique, Stephen J Pettitt, Ger Kelly, Nina Tunariu, Rachael Natrajan, Susana Banerjee, Christopher J Lord

**Affiliations:** ^1^ Division of Molecular Pathology The Institute of Cancer Research London UK; ^2^ The Breast Cancer Now Toby Robins Research Centre The Institute of Cancer Research London UK; ^3^ The Royal Marsden NHS Foundation Trust and The Institute of Cancer Research Sutton UK; ^4^ The CRUK Gene Function Laboratory and Breast Cancer Now Research Centre The Institute of Cancer Research London UK

**Keywords:** PARP inhibitor, drug resistance, cancer, BRCA2, digital droplet PCR

## Abstract

Development of resistance to platinum and poly(ADP‐ribose) polymerase inhibitors via secondary *BRCA* gene mutations that restore functional homologous recombination has been observed in a number of cancer types. Here we report a case of somatic *BRCA2* mutation in a patient with high grade serous ovarian carcinoma. A secondary mutation predicted to restore the *BRCA2* open reading frame was detected at low frequency (2.3%) in whole exome sequencing of a peritoneal biopsy at disease progression after treatment that included carboplatin and olaparib. We used digital droplet PCR (ddPCR) to verify the presence and frequency of this mutation in the biopsy sample at progression and also used this approach to assess the presence of the secondary mutation in preceding biopsies at diagnosis and first relapse. We found no evidence for the secondary mutation being present prior to the final progression biopsy, suggesting that this mutation was acquired late in the course of treatment. ddPCR provides a sensitive and specific technique to investigate the presence of low frequency mutations in a time series of biopsies.

## Introduction

Despite a proportion of women having a good initial response to platinum‐based chemotherapy, the majority of women with advanced ovarian cancer relapse and eventually die from their disease 1, 2. Epithelial ovarian cancer treatment has evolved following the regulatory approval of a number of poly(ADP‐ribose) polymerase (PARP) inhibitors 1, 3, 4, 5, 6. PARP inhibitor efficacy may extend beyond those with germline *BRCA1* or *BRCA2* mutations to a wider group of patients, with up to 50% of high‐grade serous ovarian carcinoma (HGSOC) patients suspected of having tumour‐specific homologous recombination (HR) deficiency 7, 8. Germline or somatic *BRCA* mutations could explain around 20% of these cases, with epigenetic inactivation of *BRCA1* accounting for a further 5–20%. A range of mutations in other DNA repair genes are likely to account for the remaining HR deficient cases, the most well characterised of which are *RAD51C, RAD51D, PALB2*, the Fanconi anaemia core complex and *ATM* 9, 10.

Patients whose disease progresses on PARP inhibitors or platinum therapy have provided insights into resistance mechanisms to these drugs. There have been a number of case reports describing secondary mutations that restore the function of the HR gene. In ovarian cancer, such mutations have been observed after progression on a PARP inhibitor for germline *BRCA1, RAD51C* and *RAD51D* mutations 11, 12, as well as in a case of somatic *BRCA1* mutation 11. Similar secondary mutations have been observed in platinum refractory ovarian cancer patients 12, 13, 14, 15, 16, 17, 18 and in other tumour types 18, 19, 20, 21, 22, 23, 24, 25, 26, 27. Here, we describe the case of a patient with HGSOC who received olaparib in the maintenance setting for relapsed disease after identification of a somatic *BRCA2* mutation.

## Materials and methods

### Clinical samples

The patient gave written informed consent for the use of her material for research purposes and tissue samples were obtained with appropriate ethical approval under the Royal Marsden Hospital (RMH) NHS Foundation Trust study: CCR3705 ‘Analysis of tumour specimens for biomarkers in gynaecological cancers’. All samples were reviewed at RMH and appropriate FFPE tissue blocks were selected from histology reports. Five sections (8 μm) were cut for DNA extraction. Tumour content was confirmed by a pathologist and (for the 2011 diagnostic samples) macro‐dissected as appropriate. We have analysed and reported all biopsy samples that were available from the patient.

Progressive disease was defined using RECIST 1.1 criteria as more than a 20% increase in the sum of diameters of target lesions and an absolute increase of at least 5 mm in a target lesion, from baseline to subsequent scan assessments.

### Clinical sequencing

Clinical sequencing was performed as part of the RMH NHS Foundation Trust Stratified Medicine Programme (CRUK) and Mainstreaming Genetics Programme 28, 29. *BRCA1/2* somatic mutation testing used an Illumina TruSeq custom (Illumina Inc., San Diego, CA, USA) 185‐amplicon panel targeting all coding regions and intro‐exon boundaries of *BRCA1* and *BRCA2*. Samples were sequenced on a MiSeq instrument to a target depth of ×2000 on‐target reads. Analysis was carried out using Variant Studio, reporting only Class 4/5 (pathogenic/likely pathogenic) variants.

### Exome sequencing

DNA was extracted from micro‐dissected Nuclear Fast Red stained sections using the QiaAmp DNA FFPE Tissue kit (diagnostic samples) or using the Qiagen AllPrep microkit (Qiagen GmbH, Hilden, Germany) for the fresh frozen peritoneal progression sample. Exome libraries were prepared using the Agilent SureSelectXT v6 Exome kit (Agilent Technologies, Santa Clara, CA, USA) and sequenced using 100 bp paired end reads at a target median depth of ×200. An average of 81% of reads obtained were on‐target (range 80.1–81.9). Median genome‐wide coverage achieved was ×153 for the 2017 progression sample and ×130 (range 124–135) for the diagnostic samples. The percentage of sites with at least ×40 coverage was 95% in the progression sample and 89–92% in the diagnostic samples.

### Droplet digital PCR

The primers and probes used are given in Table 1. Bio‐Rad ddPCR Supermix for Probes was used to set up the PCR. PCR conditions were: 98 °C for 10 min, 40 cycles of 94 °C for 30 s/58 °C for 2 min. Droplets generated with the Bio‐Rad QX100 Droplet Generator were analysed using the Bio‐Rad QX200 Droplet Reader and QuantaSoft software (BioRad, Hercules, CA, USA).

**Table 1:** Primers and probes for ddPCR

Probe/primer	Sequence (5′–3′)
Breakpoint probe	/5HEX/TGCAGCCATTAAATTGTCCATATCT/3IABkFQ/
Secondary mutation probe	/56‐FAM/AGCCATTAAATTGTCCACCTGCA/3IABkFQ/
Forward primer	TGCATACCCACAAACTGTAAATGA
Reverse primer	TGAAACACAAACGATTTTACCACTG

3IABkFQ, 3′ Iowa Black FQ (IDT) quencher; 56‐FAM, 5′ 6‐carboxyfluorescein; 5HEX, 5′‐hexachlorofluorescein.

### Data availability

Sequence data are deposited at the ENA, accession number: PRJEB29279. All other data are available from the corresponding author on request.

## Results

### Patient details

**Figure 1:**
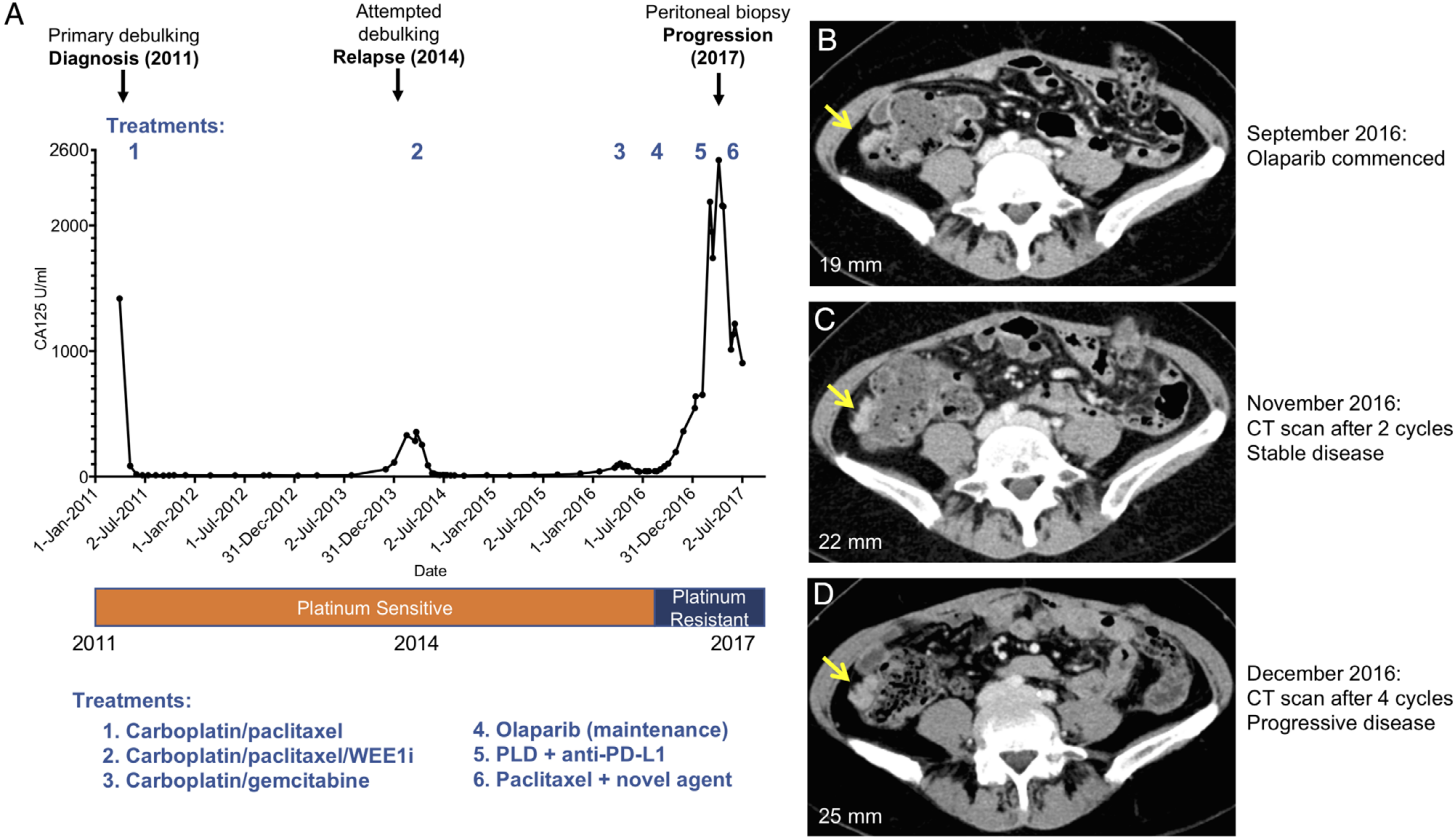
Clinical treatment course showing CA125 response. (A) Clinical treatment course and CA125 response with surgical specimens annotated above and with treatment sequence (Table 2) and platinum sensitivity status below. (B)–(D) Axial contrast enhanced CT studies performed at baseline (B), after two cycles (C) and four cycles (D) of olaparib. The yellow arrow indicates the right iliac fossa peritoneal lesion along the ascending colon that was subsequently biopsied in April 2017, with maximum diameter measurements of the peritoneal target lesion annotated on corresponding images. RECIST 1.1 assessments of the disease were SD between September and November 2016 (16% increase in overall tumour burden) and disease progression between September and December 2016 (66% increase in overall tumour burden).

A 52‐year‐old female presented with lower abdominal discomfort, bloating and a raised Cancer Antigen 125 (CA125) of 1418 U/ml. She had no significant past medical history or family history of malignancy. A CT scan identified a large pelvic mass measuring 11 × 9 × 12.6 cm arising from the right adnexa, a mixed solid‐cystic mass arising from the left ovary measuring 5.1 × 3 × 3.6 cm with peritoneal deposits, and bilateral iliac node enlargement. Her treatment history is summarised in Table 2. The patient underwent primary debulking surgery where complete cytoreduction was achieved. Histological assessment showed a high grade serous adenocarcinoma, FIGO stage IIIC. The CA125 level prior to commencement of adjuvant chemotherapy was 19 U/ml. The patient received six cycles of adjuvant three weekly carboplatin and paclitaxel (therapy 1) with her CA125 falling to 10 U/ml (Figure 1). Germline *BRCA1/*2 testing did not identify a pathogenic mutation (Table 3).

**Table 2:** Treatment sequence

Treatment stage	Associated sample	Treatment	Number of cycles	Response (RECIST 1.1 30)
1. Primary debulking and adjuvant chemotherapy	2011 Diagnosis	Carboplatin and paclitaxel	Six cycles	No CT evidence of disease recurrence till 1 June 2014 (PFI: 27 month)
2. First relapse (platinum sensitive)	2014 Relapse	Carboplatin + paclitaxel + WEE1 inhibitor*	Five cycles (with GCSF support)	CR (PFI 20 months)
3. Second relapse (platinum sensitive)	–	Carboplatin + gemcitabine	Five cycles, day 8 gemcitabine omitted after cycle 3	PR and CA125 response (GCIG)
4. Maintenance therapy (platinum sensitive BRCA mutation positive)	–	Olaparib	Four cycles	Eight weekly scan: SD, rising CA125 (59 U/ml pretreatment rising to 197 U/ml after four cycles) not evaluable by GCIG criteria
5. Third relapse (platinum resistant)	2017 Progression	PLD and anti‐PD1 therapy	Two cycles	PD
6. Fourth relapse (platinum resistant)	–	Weekly paclitaxel ± novel agent*	Three cycles	PD

The patient completed six courses of treatment as shown.

CR, complete response; GCIG, gynaecologic cancer intergroup (reference range < 35 U/ml); GCSF, granulocyte‐colony stimulating factor; PD, progressive disease.

* Trial of novel agent is yet to be reported.

**Table 3:** Mutations tested and identified in the patient

Gene test	Regions analysed	Method	Tissue	Result
*BRAF*	Exon 15	CE‐SSCA	Ovarian primary (2011 surgery)	No mutation detected
*PIK3CA*	Exons 9 and 20	CE‐SSCA	Ovarian primary (2011 surgery)	No mutation detected
*PTEN*	Exons 1–9	CE‐SSCA	Ovarian primary (2011 surgery)	No mutation detected
*TP53*	Exons 4 to 9	CE‐SSCA	Ovarian primary (2011 surgery)	c.372C>A (p.Cys124*) g.17:7675997G>T
Germline *BRCA1*	Coding exons and splice sites	Sanger sequencing and MLPA of blood‐derived DNA	Blood	No mutation detected
Germline *BRCA2*	Coding exons and splice sites	Sanger sequencing and MLPA of blood‐derived DNA	Blood	No mutation detected
Somatic *BRCA1*	Coding exons	NGS TSCA	Ovarian primary (2011 surgery)	No mutation detected
Somatic *BRCA2*	Coding exons	NGS TSCA	Ovarian primary (2011 surgery)	c.5446_5449delCTAG (p.Ser1816Leufs*23) g.13:32339799_32339802delCTAG

Genomic annotations are given using hg38 coordinates.

CE‐SSCA, capillary electrophoresis single‐strand conformation analysis; MLPA, Multiplex Ligation Dependent Probe Amplification kit; NGS TSCA, next generation sequencing, TruSeq Custom Amplicon.

Twenty‐seven months following completion of adjuvant chemotherapy, the patient's CA125 rose to 59 U/ml. A subsequent CT scan identified a soft tissue density in the anterior abdominal wall, which measured 2.4 × 1.1 cm, and serosal liver disease. The patient underwent secondary surgery with a view to debulk. She underwent a laparotomy with extensive adhesiolysis, removal of the falciform ligament and biopsies. In view of the small disease volume and distribution of disease noted at surgery, debulking was not performed as it was not felt that complete cytoreduction could be achieved. The patient enrolled into a phase II trial of carboplatin and paclitaxel in combination with a WEE1 kinase inhibitor (Table 2, treatment 2) and exhibited a radiological complete response to treatment (RECIST v1.1), with a corresponding fall in CA125 level from 356 to 10 U/ml (GCIG complete response). After a disease‐free period of 20 months, a rise in CA125 prompted a CT scan and the patient described disease‐related abdominal symptoms which correlated with the CT evidence of peritoneal and serosal liver disease. The patient was treated with gemcitabine and carboplatin (Table 2, treatment 3). Her CA125 fell to a nadir of 40 U/ml and her end of treatment CT scan showed a maintained partial response as defined by RECIST v1.1 criteria.

Following on from this second line therapy for platinum‐sensitive relapse, *BRCA1/2* testing was performed on the primary surgical sample. This revealed a somatic *BRCA2* mutation (c.5446_5449delCTAG, p.Ser1816Leu fs*23) with a high tumour variant allele frequency (VAF, 73%) indicating likely loss‐of‐heterozygosity of *BRCA2*. In September 2016, she began maintenance olaparib treatment (Table 2, treatment 4). Her CA125 level rose whilst on olaparib (59 U/ml pretreatment rising to 197 U/ml after four cycles), although a CT scan after 2 months of treatment demonstrated stable disease (SD; RECIST v1.1; Figure 1B,C). A subsequent CT scan 6 weeks later showed progression with an increase in peritoneal and serosal disease (RECIST v1.1; Figure 1D) with the CA125 rising to 547 U/ml, and olaparib was discontinued after four cycles.

The patient subsequently entered a clinical trial of pegylated liposomal doxorubicin (PLD) combined with a PD‐L1 inhibitor (Table 2, treatment 5). Following two cycles of treatment, the patient's CA125 rose from 639 to 2188 U/ml and treatment was discontinued due to radiological progression (RECIST v1.1). A peritoneal biopsy (2017 progression) was performed prior to entry into a phase II trial which included weekly paclitaxel (Table 2, treatment 6). Although there was a reduction in CA125 from 2157 to 904 U/ml, following three cycles of weekly paclitaxel ± novel agent, imaging showed RECIST progression 30 and the patient stopped treatment. The patient developed bowel obstruction and was no longer fit enough to be considered for further treatment. She died 2 months later, 6 years following initial diagnosis.

### Detection and confirmation of a secondary mutation in BRCA2

**Figure 2:**
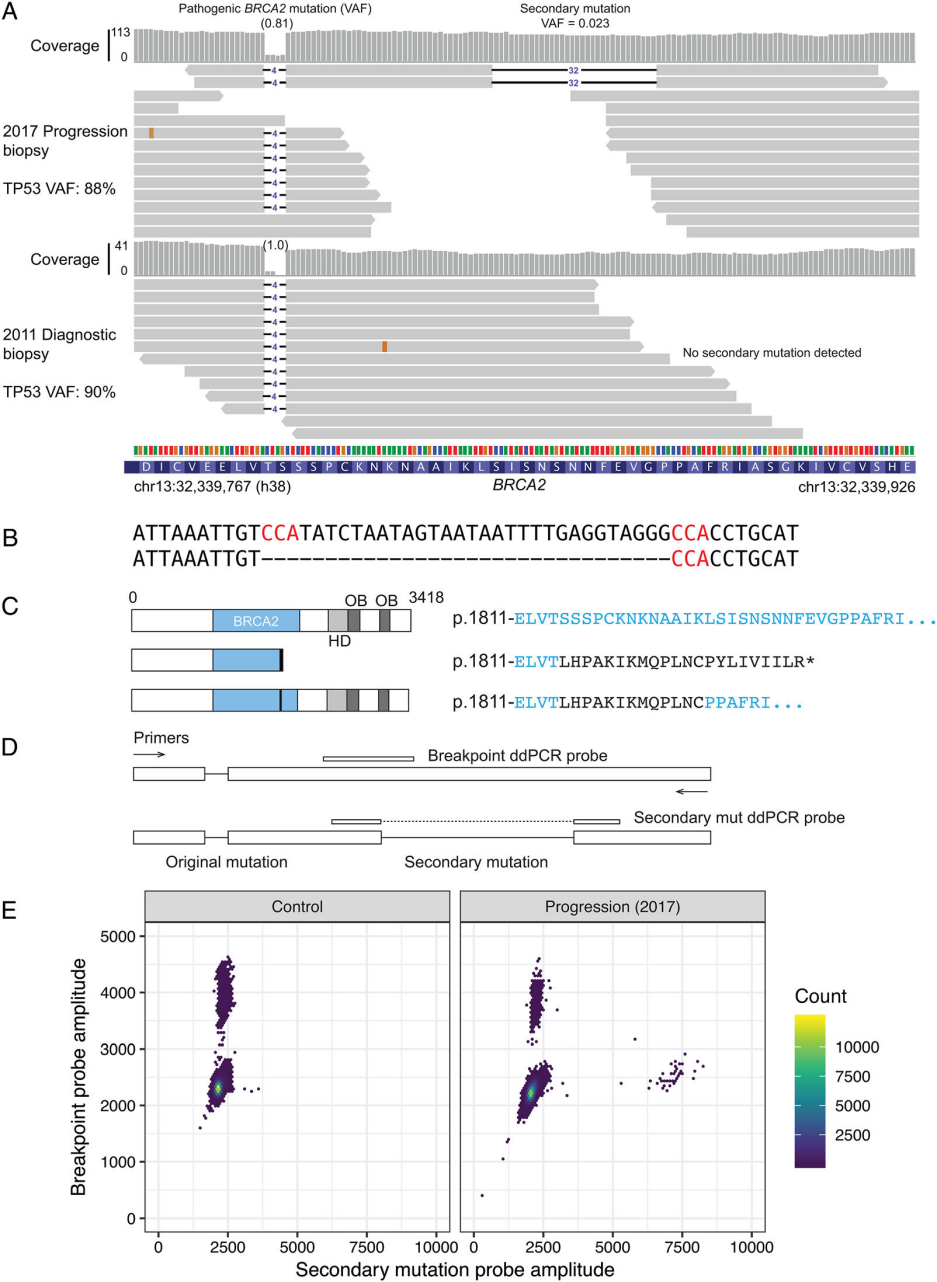
Secondary mutation restoring the BRCA2 reading frame in a peritoneal biopsy at progression. (A) Alignments of exome sequencing read to the region around the original somatic *BRCA2* mutation. Top, progression biopsy (2017, fresh frozen); bottom, diagnostic biopsy (2011, FFPE, micro‐dissected). The two reads with an additional 32 bp deletion observed in the recurrence sample are shown along with a sample of other reads. (B) DNA sequence of reference (top) and secondary mutant (bottom) at site of deletion. Flanking microhomology is highlighted in red. (C) Predicted effect of original and secondary mutations on the BRCA2 protein. HD, BRCA2 helical domain; OB, OB fold DNA binding domain. Amino acids unique to the mutant proteins are shown in black. (D) ddPCR assay design for detection of the candidate secondary mutation. (E) Scatter plots for ddPCR assay in (D). Left panel shows the CAL51 cell line as a control. Right panel shows peritoneal biopsy at progression (as in A).

Exome sequencing of DNA extracted from the 2017 progression peritoneal biopsy revealed a candidate secondary mutation in *BRCA2* (Figure 2A,C. 5489_5520delCCATATCTAATAGTAATAATTTTGAGGTAGGG) that resulted in deletion of an additional 32 bp of the *BRCA2* gene. The deleted region was flanked by microhomology, characteristic of some other previously observed intragenic deletions in *BRCA2* mutant cells 31 (Figure 2B). The net deletion in *BRCA2* was 36 bp, and was predicted to restore the native *BRCA2* open reading frame (Figure 2C). However, the secondary mutation was only represented by two out of 85 exome sequencing reads covering the deleted bases, suggesting a low allele frequency (2.3%). In each case the secondary mutation read also included the original 4 bp deletion mutation, indicating that the 32 bp deletion occurred on the same allele (Figure 2A). The VAFs of the pathogenic 4 bp deletion in *BRCA2* and the *TP53* mutation were 81 and 88% respectively, indicating a high tumour content in the biopsy.

**Figure 3:**
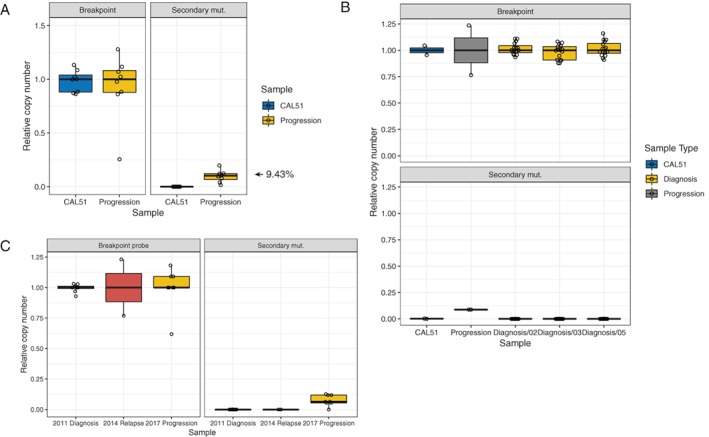
Investigation of secondary mutation frequency by ddPCR. (A) Relative copy number of reference sequence (breakpoint) and secondary mutation determined by ddPCR in CAL51 cell line DNA (control) and peritoneal progression biopsy. Data are normalised to the median breakpoint value for each sample. Each point represents one ddPCR assay well with 10 ng input genomic DNA. (B) Determination of breakpoint (top) and secondary mutation (bottom) frequencies in DNA extracted from three separate diagnostic blocks. The secondary mutation is not detected in any of the diagnostic samples. Each point represents one ddPCR assay with 60 ng input genomic DNA. (C) ddPCR assay did not detect the secondary mutation in a biopsy from the patient's first relapse in 2014.

Despite the low allele frequency and read coverage observed in the exome sequencing, the characteristics of this mutation suggested that it could represent a real reversion event. We therefore confirmed the presence of the secondary mutation using a ddPCR assay specific for the 32 bp mutation (Figure 2D,E). The mutation frequency in the 2017 progression biopsy measured by ddPCR was 9.4% (67 reversion events and 642 WT breakpoint events), compared to 2.3% based on exome sequencing coverage (Figure 3A). The original *BRCA2* mutation was detected at a frequency of 83% using a separate ddPCR probe, confirming a high tumour content in the biopsy.

### No evidence for BRCA2 secondary mutation in earlier biopsies

We also applied this ddPCR assay to DNA from the original diagnostic biopsies using a total of 1 μg DNA (approximately 100 000 diploid genome equivalents) from each of three different sections of tumours in the omentum and ovary. These three samples had average tumour content of 86% (range 73–100%) based on the original *BRCA2* VAF and 84% based on *TP53* mutation VAF (range 81–92%). We detected only five positive events compared to 62 541 WT breakpoint events (Figure 3B). The corresponding VAF across all samples (8 × 10^−5^) is below the detection limit of the ddPCR assay, suggesting that the secondary mutation was not pre‐existing at the diagnosis stage and emerged later in treatment. A limited amount of material was available from an additional biopsy from surgery at the patient's first relapse in 2014. We were able to extract DNA and analyse the presence of the secondary mutation by ddPCR. This failed to detect the secondary mutation (Figure 3C), suggesting that it was acquired after the initial relapse.

## Discussion

This observation of a secondary mutation restoring the open reading frame of an HR gene (*BRCA2*) in an ovarian cancer patient progressing on olaparib adds to the other cases reported in the literature 11, 12. The loss of heterozygosity and acquisition of a secondary mutation restoring the open reading frame suggest that this somatic *BRCA2* mutation (c.5446_5449delCTAG) was the driver for tumour formation (i.e. is a pathogenic mutation) and the cause of the initial platinum sensitivity. Both germline and somatic *BRCA2* mutations are highly predictive of sensitivity to platinum 32. We only detected the reversion event at subclonal frequencies, but found no evidence for multiple reversion events as observed in other studies 11, 18, 22, 23, 24, 27. This may be due to insufficient sequencing depth in our exome sequencing or sampling bias when taking the final recurrence biopsy. However, there are clearly many cells that lack this particular reversion, so it is interesting to speculate whether the bulk of the tumour cells would be expected to lack HR activity at this stage of the treatment – the frequency of the pathogenic *BRCA2* and *TP53* mutations was still high (81 and 88% respectively), suggesting that most cells would retain loss of function of these genes. We did not observe any genetic alterations in other known loss‐of‐function mechanisms of PARP inhibitor resistance, such as *PARP1*. There may be other genetic or epigenetic forms of drug resistance mechanisms in other cells (e.g. overexpression of drug efflux pump proteins) that were not detected by the sequencing done here. Although reversions have been strongly associated with resistance to platinum and PARP inhibitors 33, it is possible that the reversion mutation observed here has emerged as a passenger event and is not responsible for the treatment resistance. It is also possible that the clonal architecture of the tumour has changed in the intervening period between stopping olaparib and acquisition of the biopsy, particularly if the secondary mutant cells have a fitness defect compared to cells with the original mutation in the absence of PARP inhibitor treatment.

Another unresolved question about reversion mutations is whether they arise early in disease progression and are selected in a Darwinian fashion by platinum and/or PARP inhibitor treatment, or later in the course of disease, possibly even caused by DNA damage associated with the treatment itself. Longitudinal studies of individual patients using sensitive detection methods such as ddPCR could potentially address this question. We applied the ddPCR assay that we developed to an earlier biopsy taken at surgery after the patient's first relapse in 2014, as well as to a number of spatially distinct diagnostic samples. We could not confidently observe any reversion events in these experiments. In the absence of a positive result, we can conclude that either the reversion is absent, sampling was not adequate or the reversion is present at levels below the limit of the assay.

## Author contributions statement

SJP, SK, SB and CJL designed the study. RN, CJL and SB supervised the study. SK acquired samples and analysed the clinical data. NT reviewed the radiology. SJP analysed data, processed samples and designed the ddPCR. GK processed samples and carried out ddPCR experiments. SJP, SK, SB and CJL wrote the paper.
